# Cardiovascular and all-cause mortality attributable to loneliness in older Swedish men and women

**DOI:** 10.1186/s12877-020-01603-x

**Published:** 2020-06-09

**Authors:** Masuma Novak, Margda Waern, Lena Johansson, Anna Zettergren, Lina Ryden, Hanna Wetterberg, Pia Gudmundsson, Ingmar Skoog

**Affiliations:** Institute of Neuroscience and Physiology, Department of Psychiatry and Neurochemistry, Unit of Psychiatric Epidemiology, Sahlgrenska Academy, University of Gothenburg, Wallinsgatan 6, 431 41 Mölndal, Sweden

**Keywords:** Loneliness, CVD, Mortality, Sweden, Epidemiology

## Abstract

**Background:**

This study examined whether loneliness predicts cardiovascular- and all-cause mortality in older men and women.

**Methods:**

Baseline data from the Gothenburg H70 Birth Cohort Studies, collected during 2000 on 70-year-olds born 1930 and living in Gothenburg were used for analysis (*n* = 524). Mortality data were analyzed until 2012 through Swedish national registers.

**Results:**

Perceived loneliness was reported by 17.1% of the men and 30.9% of the women in a face-to-face interview with mental health professional. A total of 142 participants died during the 12-year follow-up period, with 5334 person-years at risk, corresponding to 26.6 deaths/1000 person-years. Cardiovascular disease accounted for 59.2% of all deaths. The cumulative rates/1000 person-years for cardiovascular mortality were 20.8 (men) and 11.5 (women), and for all-cause mortality 33.8 (men) and 20.5 (women), respectively. In Cox regression models, no significant increased risk of mortality was seen for men with loneliness compared to men without loneliness (cardiovascular mortality HR 1.52, 95% CI 0.78–2.96; all-cause HR 1.32, 95% CI 0.77–2.28). Increased risk of cardiovascular mortality was observed in women with loneliness compared to those without (HR 2.25 95% CI 1.14–4.45), and the risk remained significant in a multivariable-adjusted model (HR 2.42 95% CI 1.04–5.65).

**Conclusions:**

Loneliness was shown to be an independent predictor of cardiovascular mortality in women. We found no evidence to indicate that loneliness was associated with an increased risk of either cardiovascular- or all-cause mortality in men.

## Background

Loneliness is a subjective, negative emotional state, generally defined as perceiving less social contacts than desired [1]. Loneliness may be experienced by people of all ages, however, with advancing age, social contacts are reduced due to factors, such as losses of spouse or friends or mobility impairments, resulting in a higher prevalence of loneliness in elderly populations [2]. A large body of evidence suggests that loneliness is a major risk factor for poor mental and physical health in later life [3]. For instance, feeling of loneliness is reported to be associated with increased risks of cardiovascular diseases (CVD), depressive symptoms, impaired cognitive performance, and dementia [3]. Although there is considerable literature on the impact of loneliness on morbidity, there is modest data on its effect on mortality [3]. These studies have reported higher rates of mortality among individuals with loneliness [4–11]. However, findings across these studies are inconsistent on whether loneliness independently predicts mortality risk after adjusting for initial health and social factors. The aim of the present study was to examine whether loneliness independently predicts cardiovascular- and all-cause mortality in older people after adjusting for initial health status, health behaviors, depression, living alone and economic situation.

## Methods

### Study population

The Gothenburg H70 Birth Cohort Studies (the H70 studies) are ongoing population-based longitudinal studies of health and ageing. Full details of these studies have been reported elsewhere [12–16]. In brief, initiated in 1971, the H70 studies are a series of cohort studies of older men and women living in Gothenburg, Sweden. Seventy-year-old men and women listed in national population registers in Gothenburg were systematically selected based on their birth dates and they underwent extensive medical, social, psychiatric, and physical examinations.

For the purpose of the present study, baseline data on the 1930 birth cohort collected during 2000 was used (*n* = 524, response rate 70%). Forty-seven percent of non-participants was surveyed for a shorter health interview. Participants and non-participants were similar regarding self-rated health, history of myocardial infarction, diabetes or smoking status, but married men were significantly overrepresented among participating men [17].

### Assessment of loneliness

Self-perceived feeling of loneliness was assessed by a single question as ‘do you feel lonely?’ There were four alternative responses where 1 indicated never feeling lonely, 2 seldom, 3 sometimes, and 4 very often (see Table 1 for sample distribution). The four categories were then merged into a dichotomous variable as 0 = not lonely (responses 1–2), and 1 = lonely (responses 3–4).

**Table 1:** Number and percentages of men and women in each of the four response categories of self-perceived feelings of loneliness

	All N	Never (response 1) n (%)	Seldom (response 2) n (%)	Sometimes (response 3) n (%)	Often (response 4) n (%)
**Men**	240	147 (61.3)	52 (21.7)	39 (16.3)	2 (0.8)
**Women**	272	104 (38.2)	84 (30.9)	70 (25.7)	14 (5.1)
**All**	512	251 (49.0)	136 (26.6)	109 (21.3)	16 (3.1)

### Mortality

Based on unique personal identification numbers and using the Swedish national registers (the national population register and the national cause of death register), cohorts were followed for 12 years from the date of their baseline examination or until death. Cardiovascular deaths were those with International Classification of Diseases, 10th Revision (ICD-10) codes I.00-I.99.

### Other covariates

Adverse socioeconomic status, health and health related behavioral factors that have previously been shown to be associated with loneliness [18–20] were included as possible confounding factors. Current perceived economic situation was assessed using a seven-point scale ranging from excellent to very bad (coded from 1 to 7). The seven-point scale was then merged into three categories: good (scale points 1–3, excellent, very good, good), Average (scale point 4), and poor (scale points 5–7, not very good, bad, very bad). Living alone was categorized as individuals who are single, or divorced, or widowed and live alone versus individuals who live with a partner (married/cohabiting/having partner but lives separate or live-apart-together or occasionally live together, Swedish term is ‘*särbo’*) or with someone else. Smoking status was categorized as current smoker (regular or occasional), previous smoker, and never smoker. Leisure time physical activity was defined as moderate/regular versus inactive. Alcohol consumption was measured with questions regarding weekly consumption of beer, wine, and spirits in centiliters [21] during the past month. Based on these volumes, average weekly grams of alcohol consumption were calculated using conversion factors based on average alcohol concentration by volume (spirits 1 cl = 3 g, wine 1 cl = 1 g, beer > 3.5% 1 cl = 1/3 g). Body mass index (BMI) was calculated from measured weight and height (weight in kg/height in m^2^). Previous history of having (yes/no) cancer, diabetes, coronary heart disease and stroke was based on self-report as well as from medical examinations conducted by a study physician. Systolic and diastolic blood pressure (SBP, DBP) were measured in the sitting position after a minimum of 5 min of rest. Blood samples were drawn from an antecubital vein and serum triglyceride measurement was determined according to standard laboratory procedures. ADL disability was defined based on a six-item scale of activities of daily living (ADL). The ADL scale measured self-reported difficulties in performing daily life activities including transferring, dressing, bathing, using toilet, eating, and continence. Each item was coded as 0 = no need of help from another person, and 1 = need help. A composite index was created by summing up all the six items ranging from 0 to 6 (need no help to need help in all six activities). The index was then dichotomized as 0 (no ADL disability) and 1 (ADL disability, scale 1–6). Based on symptoms elucidated during a psychiatric examination, major depression was diagnosed according to the DSM-5 criteria (American Psychiatric Association) [22], and minor depression according to the DSM-IV research criteria [23]. Definition of these variables has been described previously [24].

### Statistical analysis

Using descriptive statistics, differences in the distribution of baseline characteristics in men and women according to their loneliness status were examined using the Pearson x^2^-test for categorical variables and Student’s t-test for continuous variables. Descriptive statistics are presented as percentages or mean values with standard deviations (SD). All P-values are two-sided and values of < 0.05 were considered statistically significant. The survival function for the 12-year period according to loneliness status was assessed using the Kaplan Meier method, and the log-rank test was used to evaluate group differences. Cox proportional hazard regression models were used to study the association between loneliness status at baseline and cardiovascular and all-cause- mortality during 12-year follow-up. Both unadjusted and multivariable adjusted regressions were carried out separately for cardiovascular- and all-cause mortality. Factors that were shown to be associated with loneliness were included in multivariable models, where all the selected variables were entered simultaneously. Estimates derived from Cox regressions are presented in graphical format showing hazard ratios [21] and 95% CI. Statistical analyses were performed using SPSS, Windows version 25.0 (SPSS Inc., Chicago, IL, USA) and graphics were produced using R version 3.4.3 (The R Foundation for Statistical Computing).

## Results

The current analyses were conducted on 512 participants (240 men, 272 women) after exclusion of 12 individuals with missing data on loneliness. A total of 125 (24.4%) participants reported being lonely at baseline. The prevalence of loneliness was 17.1% (*n* = 41) in men and 30.9% (*n* = 84) in women (*p = 0.001*).

### Loneliness and background characteristics

Table 2 presents background characteristics of men and women according to their loneliness status. Men with loneliness reported more often poor economic status, living alone, and physical inactivity and were more often diagnosed with depression than men with no loneliness. No significant differences were observed regarding smoking, alcohol consumption, BMI, and history of any health related variables. Women with loneliness more often reported poor economic status, chronic bronchitis, and living alone, and were more often diagnosed with depression than women without loneliness. No significant differences were observed regarding smoking, physical activity, alcohol consumption, BMI, or history of other health related variables.

**Table 2:** Distribution of baseline characteristics among men and women according to their perceived loneliness status (*N* = 512)

	Men ***N*** = 240			Women ***N*** = 272		
	Lonely ***N*** = 41	Not lonely ***N*** = 199	***p***-value	Lonely ***N*** = 84	Not lonely ***N*** = 188	***p***-value
**Current perceived economic situation**						
Good	47.5 (19)	77.7 (150)		48.8 (39)	72.2 (132)	
Average	27.5 (11)	15.6 (30)		38.8 (31)	17.8 (32)	
Poor	25.0 (10)	6.7 (13)	0.000	12.5 (10)	10.0 (18)	0.001
**Living alone**	52.5 (21)	9.1 (18)	0.000	60.8 (48)	39.2 (71)	0.001
**Smoking status:**						
Never smoker	22.0 (9)	33.3 (66)		51.2 (42)	62.5 (115)	
Previous smoker	53.6 (22)	53.5 (106)		25.6 (21)	23.9 (44)	
Current smoker	24.4 (10)	13.2 (26)	0.119	23.2 (19)	13.6 (25)	0.109
**Physical activity:**						
Regular	38.5 (15)	64.4 (125)		32.5 (25)	35.8 (63)	
Inactive	61.5 (24)	35.6 (69)	0.003	67.5 (52)	64.2 (113)	0.609
**BMI**	27.13 ± 3.49	26.88 ± 3.95	0.704	26.63 ± 4.04	27.22 ± 4.69	0.330
**Alcohol (gm/per week)**	51.18 ± 64.61	76.29 ± 108.27	0.071	25.69 ± 31.14	34.03 ± 52.14	0.228
**Health related variables:**						
SBP (mm Hg)	151.97 ± 22.46	157.71 ± 20.40	0.146	151.43 ± 23.85	153.70 ± 22.07	0.504
DBP (mm Hg)	84.76 ± 8.94	87.81 ± 9.95	0.103	85.40 ± 11.85	84.36 ± 10.47	0.517
Triglyceride (mmol/l)	1.30 + 0.54	1.39 ± 0.57	0.386	1.40 ± 0.56	1.41 ± 0.85	0.995
Diabetes	12.2 (5)	11.2 (22)	0.850	11.5 (9)	9.0 (16)	0.536
CHD	17.1 (7)	10.7 (21)	0.246	5.0 (4)	5.5 (10)	0.877
Stroke	2.4 (1)	5.1 (10)	–	9.8 (8)	7.0 (13)	0.445
Cancer	12.2 (5)	15.3 (30)	0.610	18.2 (14)	12.6 (22)	0.248
Chronic bronchitis	19.5 (8)	13.2 (26)	0.293	25.6 (20)	15.2 (27)	0.046
Arthritis	17.1 (7)	17.3 (34)	0.966	34.6 (27)	31.8 (56)	0.661
ADL disability	17.9 (7)	9.2 (17)	0.108	14.9 (11)	10.6 (18)	0.343
**Depression:**						
No	68.4 (26)	95.7 (180)		61.3 (49)	92.8 (168)	
Minor	21.1 (8)	4.3 (8)		25.0 (20)	5.5 (10)	
Major	10.5 (4)	0	0.000	13.8 (11)	1.7 (3)	0.000

Values presented in this table are mean ± SD or percentage with number of subjects in parenthesis

*BMI* Body mass index (wt in kg/ht. in m^2^), *SBP* Systolic blood pressure, *DBP* Diastolic blood pressure, *CHD* Coronary heart disease

### Gender, loneliness and mortality

A total of 142 (27.7%) participants died during the 12-year follow-up period, with a median follow-up of 11 years and 5334 person-years at risk, corresponding to 26.6 deaths per 1000 person-years. Cardiovascular disease accounted for 59.2% (*n* = 84) of all deaths, which corresponds to 15.8 deaths per 1000 person-years. The cumulative rates per 1000 person-years for cardiovascular mortality were 20.8 (men) and 11.5 (women), and for all-cause mortality were 33.8 (men) and 20.5 (women), respectively.

**Fig. 1:**
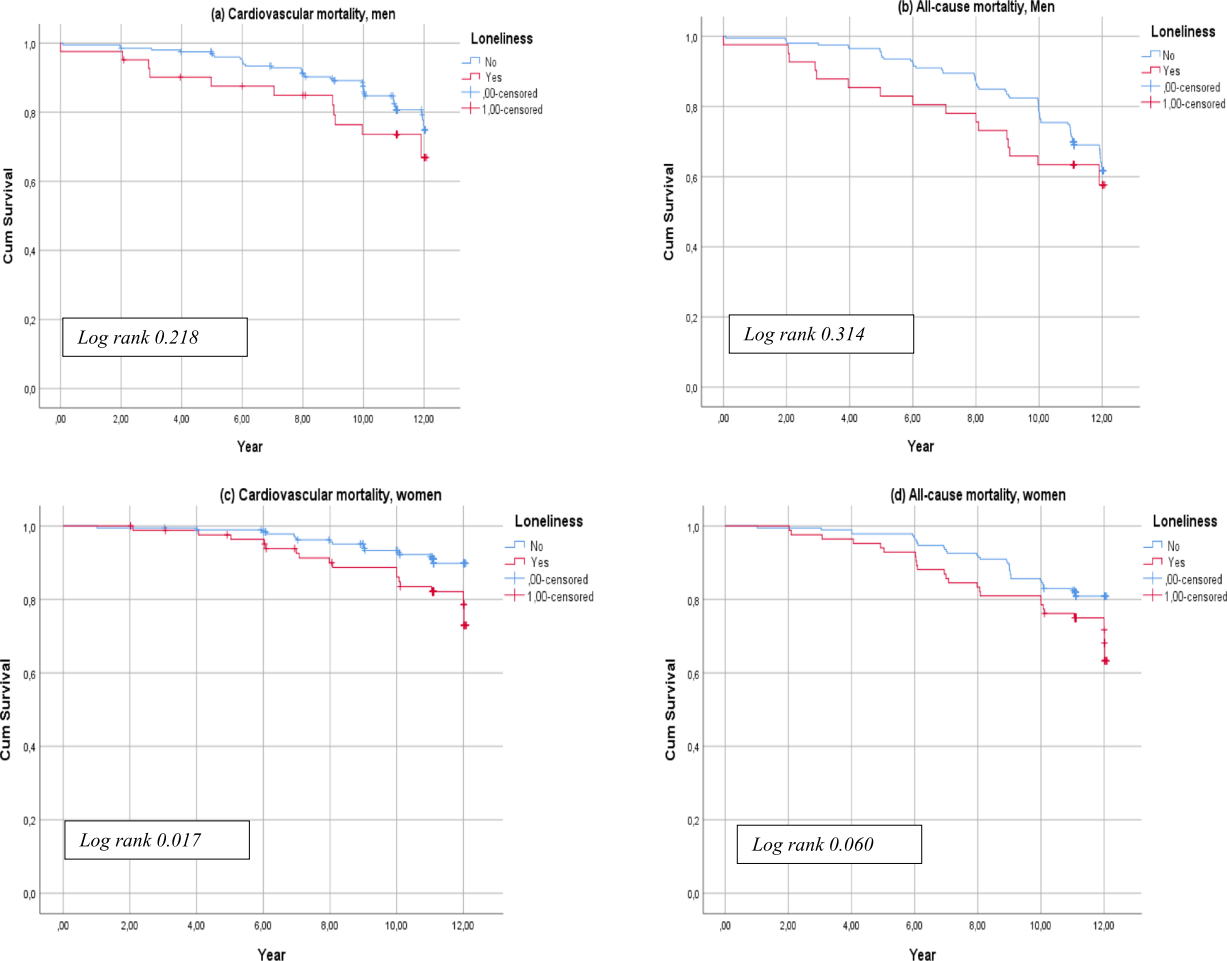
Kaplan-Meier survival estimates for 12-years follow-up according to perceived loneliness status for (**a**) cardiovascular mortality, men; (**b**) all-cause mortality, men; (**c**) cardiovascular mortality, women; and (**d**) all-cause mortality, women

Kaplan Meier analysis showed no significant difference in survival between men with and without loneliness for either cardiovascular- or all-cause mortality (log rank*, p > 0.05*) (Fig. 1a and b). For women, Kaplan Meier curves showed no significant difference in all-cause mortality by loneliness status (Fig. 1c), but a lower survival rate was observed among women with loneliness compared to women with no loneliness regarding cardiovascular mortality (log rank*, p = 0.017*) (Fig. 1d).

**Fig. 2:**
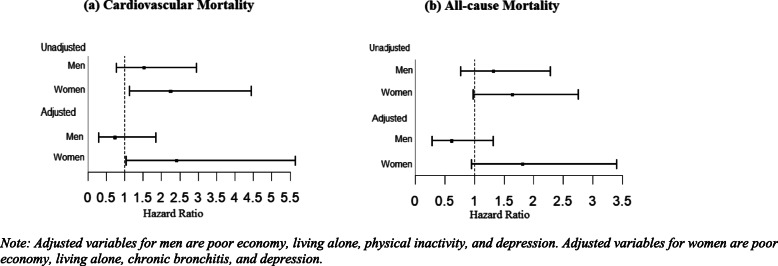
Cox proportional hazard ratios (HR) and 95% confidence intervals (CI) of (**a**) cardiovascular mortality and (**b**) all-cause mortality in men and women with loneliness. Note: Adjusted variables for men are poor economy, living alone, physical inactivity, and depression. Adjusted variables for women are poor economy, living alone, chronic bronchitis, and depression

Figure 2 presents HRs and 95% CI of (a) cardiovascular and (b) all-cause mortality according to gender and loneliness status. Similar to Kaplan Meier results, no significant increased risk of mortality was observed for men with loneliness compared to men with no loneliness for both cardiovascular (HR 1.52 95% CI 0.78–2.96) and all-cause mortality (HR 1.32 95% CI 0.77–2.28). In all-cause mortality, no significant increased risk of mortality was seen for women with loneliness compared to women with no loneliness (HR 1.64 95% CI 0.98–2.76). However, women with loneliness had significantly higher risks of cardiovascular mortality compared to women with no loneliness (HR 2.25 95% CI 1.14–4.45). The high risks of cardiovascular mortality in women remained significant in the multivariable-adjusted model (2.42 95% CI 1.04–5.65).

## Discussion

This study examined if loneliness at age 70 was prospectively associated with cardiovascular- and all-cause mortality over a 12-year follow-up period, after controlling for initial health status, health behaviors, depression, and other social factors. We found that loneliness was an independent predictor of cardiovascular mortality in women, while there was no significant association with all-cause mortality. Among men, we found no evidence of an association between loneliness and subsequent death whether cardiovascular- or all-cause both in unadjusted or multivariable-adjusted models.

The strengths of the study included the population-based samples of both men and women and the comprehensive examinations data on socio-demographic factors, medical history, and clinical- and physical measurements, including loneliness status. However, this study also had a number of limitations. First, although the response rate at baseline was 70%, which is higher than in many other studies [25], we cannot exclude the possibility that participants were healthier than non-participants. Although a short health survey in non-participants revealed that participants and non-participants were similar in terms of health history and health related behaviors, married men were overrepresented among participating men [17]. This may imply that lonely men are underrepresented in the sample, as unmarried status is strongly associated with loneliness [19], which may have caused selection bias in our study. Another limitation was the definition of loneliness, which was assessed with a single-item question. This may result in underreporting due to the stigma associated with being identified as lonely [2, 26]. Therefore, this single measurement may not capture the overall influence of loneliness on mortality. This single item question of loneliness, however, is most common and a widely used measure [3], which previously has been shown to predict mortality [5, 7, 9].

As expected, feeling lonely was more common in women than in men. A recent review concluded that women are more likely to report loneliness than their male counterparts regardless of country studied and the classification of loneliness used [19]. One possible explanation for the greater loneliness experienced by older women may be that they are more willing to admit socially unacceptable feelings than men [1], and that disclosing loneliness may be more socially accepted in women than in men [27].

Findings across previous studies on loneliness and all-cause mortality are inconsistent as to whether loneliness independently predicts mortality risk after adjusting for initial health status, health behaviors, depression, and other social factors. The lack of association between loneliness and all-cause mortality found in our present study is similar to several previous studies conducted in Amsterdam [11], France [7], England [10], and China [5]. Two studies conducted in the USA, however, reported that loneliness was an independent predictor of all-cause mortality after adjusting for prior health and health behavior and depression [4, 8]. Similarly, a recent meta-analysis reported that loneliness is associated with all-cause mortality in both gender but this effect is slightly stronger in men than in women [28]. On the other hand, the Amsterdam study of the elderly (AMSTEL) on 4004 older men and women aged 65–84 years have shown that loneliness is an independent risk factor for all-cause mortality in men but not in women [29]. These inconsistent findings across studies may be related to varying study designs, sample size, methods and follow-up periods, but also differences in cultural settings.

Studies concerning long-term longitudinal associations between loneliness and cardiovascular death are scarce. To our knowledge, our study is the 2nd to examine loneliness in relationship to cardiovascular death in a population-based sample of men and women. The first study was conducted using UK biobank data on 466,901 men and women by Elovainio M, *et. al.* and reported that loneliness was not independently associated with cardiovascular mortality for both genders in multivariable adjusted models [6]*.* Our study result partly contradicts the study by Elovainio M, *et. al.,* as we observed an independent association between loneliness and cardiovascular mortality in women but not in men. Possible mechanisms by which loneliness contribute to cardiovascular mortality, which is observed in our study, may be that loneliness affects cardiovascular health by altering biomarkers and shaping health behavior that are associated with increased CVD risks. For example, loneliness has been associated with elevated blood pressure [18], elevated triglycerides level [20], CHD [30], smoking, and physical inactivity [19]. In our study, no associations were found in women between loneliness and baseline SBP, DBP, CHD, triglyceride level or most of the prior health variables including smoking and physical activity. However, women with loneliness more often reported poor economic status, chronic bronchitis, and living alone, and were more often diagnosed with depression, compared to those without loneliness. Loneliness predicted cardiovascular mortality in women after adjusting for the associated factors suggesting that loneliness alters physiology at a more fundamental level. Future research should include efforts to examine how physiological processes contribute to the effect of loneliness on mortality.

## Conclusions

Loneliness was an independent predictor of cardiovascular mortality in women, while there was no evidence to indicate that loneliness was associated with an increased risk of either cardiovascular- or all-cause mortality in men. Our results emphasizes the importance of considering women with loneliness as a high-risk group to target for public health and medical care efforts in reducing cardiovascular mortality.

## Data Availability

The data supporting this article can be made available from the corresponding author on reasonable request.

## Abbreviations

CVD: Cardiovascular disease
CHD: Coronary heart disease
ICD: International Classification of Diseases
BMI: Body mass index
SBP: Systolic blood pressure
DBP: Diastolic blood pressure
ADL: Activities of daily living
SD: Standard deviations
CI: Confidence interval
